# Effects of Neurodynamics Along With Conventional Exercises on Sciatica Patients: A Single-Blinded Randomized Clinical Trial

**DOI:** 10.7759/cureus.59722

**Published:** 2024-05-06

**Authors:** Anwesh Pradhan, Muthukumaran J

**Affiliations:** 1 Department of Physiotherapy, Saveetha College of Physiotherapy, Saveetha Institute of Medical and Technical Sciences, Chennai, IND

**Keywords:** emg biofeedback, straight leg raise, sciatica, conventional exercise, neurodynamics

## Abstract

Introduction

Sciatica refers to a pain that travels along the course of the sciatic nerve. Patients also often experience paresthesia along with the pain in thighs, which may further radiate to the legs. Most commonly, compression of the lumbosacral nerve root is the cause of this syndrome. Neurodynamics and conventional exercises are considered effective treatment procedures for sciatica. This study aims to find out the efficacy of neurodynamics along with conventional exercises and conventional exercises alone.

Methods

A total of 58 patients with sciatica aged between 30 and 60 years of both genders were included in the study and randomly divided into a neurodynamic group (n=29) and a conventional group (n=29). Pre-test data were collected before the interventions, and post-test data were collected on the 14th day. The 101 numeric pain rating scale (NPRS) was used to measure data of sciatic pain, the patient-specific functional scale (PSFS) was used to measure the health-related quality of life (HRQL), and a surface electromyography (EMG) biofeedback instrument was used to measure the peak and average muscle activation of the biceps femoris muscle.

Results

The pre-post data analysis of the neurodynamics and conventional group showed significant (p<0.05) improvement in 101 NPRS, PSFS, and peak EMG values. Insignificant (p>0.05) improvements were seen in average EMG values in the conventional group, and significant (p<0.05) improvement were seen in the neurodynamic group. Between-group analysis showed insignificant (p>0.05) differences in 101 NPRS as well as peak and average EMG values and showed significant (p<0.05) differences in PSFS values.

Conclusion

Neurodynamics with conventional exercises can help in reducing pain, improving muscle activation of the biceps femoris, and elevating the HRQL of the patient.

## Introduction

Sciatica is usually characterized by nagging pain along the course of the sciatic nerve or associated lumbosacral nerve. It is evident that patients also complain of paresthesia and weakness alongside the pain. These symptoms can affect the quality of life of the patients severely. Sciatica also can be seen in patients, along with low back pain (LBP) in most of them. Usually, compression or irritation of the nerve roots from L4 to S3 causes radiculopathy pain and paresthesia in the sciatic nerve course known as sciatica or lumbosacral radiculopathy [1]. Mostly this compression and irritation occurs due to a herniated disc at the lumbosacral region and sometimes due to piriformis syndromes [1]. Lifetime incidence of sciatica is reported to be between 10% and 40%, with an annual incidence of 1% to 5% [2]. The peak incidence is seen mostly in the fourth decade of life, with no gender dominance [2].

Physiotherapy treatments are usually considered the first line of treatment for sciatica patients [3]. Physiotherapists usually prefer to treat LBP with sciatica using various exercises and electrotherapy treatments [4]. In recent studies, neurodynamic mobilization of the sciatic nerve showed good results in reducing the symptoms, whereas conventional treatments that include various exercises and also considered by many to date showed good improvements in patients [5]. Studies also suggest that exercises and ergonomic advice can reduce the symptoms of sciatica, but most of them showed results in the long run and usually can be considered to treat to avoid or delay surgery [6]. However, a few studies also showed that early intervention with isometric exercises can affect the symptoms [7]. On the other hand, some studies also suggest that exercises alone cannot help much with sciatica [8].

Some recent studies, on the other hand, showed that a sciatic nerve mobilization is an effective approach to improving the symptoms of sciatica and improving the flexibility of the affected muscles [9]. Manual therapy such as neurodynamic mobilization is also considered an effective tool apart from other physiotherapeutic treatments by many studies [5,9]. A topical review by Ostelo reported that kinesiophobia can also be considered as an indication for exercise therapy like neurodynamic mobilization [1]. Patients with sciatica often show kinesiophobia due to the sciatic pain they experience. In this study, we tried to find out the differences between the effects of neurodynamic mobilization combined with conventional exercises and conventional exercises alone. Studies suggest the long-term application of conventional exercises to get their effects [5-7], and the application of neurodynamic mobilizations and other manipulative therapy as an alternative treatment [3,9]. The studies tried to find out the effects of the combination of neurodynamic mobilization and conventional exercises when applied for a shorter duration. The effects were checked for radicular pain and health-related quality of life (HRQL). In addition, for muscle activation of the muscle supplied by the sciatic nerve, using electromyography (EMG) biofeedback will help check the immediate effects. Therefore, the study aims to determine whether the combination of neurodynamic mobilization and conventional exercises or conventional exercises alone can effectively reduce sciatic pain, hence improving the HRQL. It also tries to find out whether these interventions can improve the muscle activation affected by sciatica.

## Materials and methods

The single-blinded randomized clinical trial was carried out after receiving clearance from the Institutional Ethics Committee of Saveetha Medical College and Hospital (003/05/2019/IEC/SMCH). The study was also registered with the Clinical Trial Registration of India (CTRI/2020/04/024554).

The patients were already assessed and diagnosed as sciatica patients by the concerned physician, mostly by clinical examinations and required investigations such as MRI and X-ray. The patients were then sent to the Physiotherapy Department for treatment. All the patients were recruited from the Physiotherapy Outpatient Department. The patients were screened again with the straight leg-raising test as per the inclusion criteria. A total of 63 patients of both genders, aged between 30 and 60 years, were included after taking informed consent from them. Out of them, five patients discontinued and 58 patients completed the protocol. They were randomly divided into a conventional group (n=29) and a neurodynamics group (n=29). Patients of both groups were taught to perform a set of exercises once a day, which they recorded in the compliance chart. Patients in the neurodynamics group addedly received gentle sciatic nerve glides in the standard straight leg raising (SLR) position. Pre-test data were collected before the interventions, and post-test data were collected on the 14th day.

Inclusion criteria

Patients of both genders within the age group of 30 to 60 years were included. Patients having positive SLR tests between 35 and 70 degrees, patients diagnosed with sciatica for the first time that was confirmed by the patient’s present and past medical history, and patients who were still active and independently ambulated were included in the study.

Exclusion criteria

Patients with acute herniated discs, those having a history of spinal trauma or spinal surgical history, and those with pregnancy were excluded from the study.

Conventional exercises

The exercises given to the patients of both groups were bridging exercises, knee-to-chest exercises, lower back extension exercises, upper back extension exercises, and cat-and-camel exercises.

All the exercises were performed in one session a day. In each session, three sets of all the exercises were performed. In each set, 10 repetitions of the active performances of exercises were done by the patients. A total of 10 sessions were performed in 14 days. Patients performed two days of exercise and took one day of rest.

Neurodynamic technique

The physiotherapist gave gentle sciatic nerve glides manually to the patients in the standard SLR position. The degree of SLR was set by the pre-intervention degree of positive SLR every session.

Five glides were given continuously, and then rest was given for one minute. Like that, the glides were performed for five minutes, which is one session. A total of 10 sessions were performed in 14 days. Sciatic nerve glides were performed for two days, and one day of rest was given.

Outcome measures

101 Numeric Pain Rating Scale

The 101 numeric pain rating scale (NPRS) measured data on sciatic pain. Patients were asked to mark their pain level on a scale of 0 to 100scale, which was evaluated before and after the interventions. Overall, 0 was considered as no pain, and 100 was considered as maximum pain felt by the patient. NPRS is a very valid and reliable scale, especially for LBP and lumbar disc herniation patients [10].

Patient-Specific Functional Scale

The patient-specific functional scale (PSFS) measured HRQL. This scale was developed by Stratford et al. It is a self-report outcome measure of function. It is a valid, reliable, and easy-to-administer scale and applies to various types of clinical presentations. Patients are to identify up to five important activities they have difficulty performing and rate them from 0 to 10, with 0 considered as no difficulty and 10 considered as maximum difficulty felt by the patient. The patient-selected values were evaluated before and after the interventions [11].

Surface EMG Biofeedback

A surface EMG biofeedback instrument was used for measuring the peak and average muscle activation of the biceps femoris muscle. EMG biofeedback using surface electrodes detects the changes in skeletal muscle activity and shows the myoelectrical signals into visual and auditory signals [12]. The peak and average activation achieved by the patient's active muscle contractions were measured and evaluated before and after the interventions.

## Results

SPSS Version 25 software (IBM Corp., Armonk, NY) was used to analyze the collected pre-post data of both groups. A paired t-test was used to conduct the within-group analysis of the 101 NPRS value, PSFS score, and peak and average muscle activation of the biceps femoris muscle. An independent t-test was used to conduct the between-group analysis of 101 NPRS value, PSFS score, and peak and average muscle activation of the biceps femoris muscle.

Descriptive analysis was conducted to describe the demographic data (Table 1). The mean age for patients in the conventional group was 48.55 ± 8.04 years and that for patients in the neurodynamic group was 48.52 ± 9.59 years. In the conventional group, 27.59% were male and 72.41% were female patients, whereas in the neurodynamic group, 34.48% were male and 65.52% were female patients.

**Table 1:** Descriptive analysis of demographic data

Treatment group	N	Mean age (years)	Gender	Percentage	Affected side	Percentage
Conventional	29	48.55 ± 8.04	Male	27.59%	Left	41.38%
			Female	72.41%	Right	58.62%
Neurodynamic	29	48.52 ± 9.59	Male	34.48%	Left	62.07%
			Female	65.52%	Right	37.93%

The pre-post data analysis of the conventional group showed significant (p<0.05) improvement in 101 NPRS and PSFS values, which shows improvement in sciatic pain and HRQL in the patients. The EMG record showed significant (p<0.05) improvement in the peak muscle activation of the biceps femoris muscle, whereas the average muscle activation of biceps femoris showed insignificant (p>0.05) changes (Table 2). This suggests that conventional exercises improved peak muscle performance but not average muscle performance.

**Table 2:** Conventional group pre-post data analysis t, t-statistics; df, degree of freedom; p-value, probability value (95% confidence interval of the difference); NPRS, numeric pain rating scale; PSFS, patient-specific functional scale *p<0.05

Variables	N	Mean difference	Standard deviation	t	df	P-value
Pre-test 101 NPRS vs post-test 101 NPRS	29	44.66	11.33	21.22	28	0.00^*^
Pre-test PSFS vs post-test PSFS	29	-13.69	7.95	-9.28	28	0.00^*^
Pre-test vs post-test muscle activation peak value	29	-59.39	142.62	-2.24	28	0.03^*^
Pre-test vs post-test muscle activation average value	29	-7.34	22.70	-1.74	28	0.09

The pre-post data analysis of the neurodynamics group also showed significant (p<0.05) improvement in 101 NPRS and PSFS values, which shows improvement in sciatic pain and HRQL in the patients. The EMG record showed significant (p<0.05) improvement in the peak muscle activation and the average muscle activation of the biceps femoris muscle. That means neurodynamics along with conventional exercises can improve both peak and average muscle performance (Table 3).

**Table 3:** Neurodynamics group pre-post data analysis t, t-statistics; df, degree of freedom; p-value, probability value (95% confidence interval of the difference); NPRS, numeric pain rating scale; PSFS, patient-specific functional scale *p<0.05

Variables	N	Mean difference	Standard deviation	t	df	P-value
Pre-test 101 NPRS vs post-test 101 NPRS	29	51.89	18.66	14.98	28	0.00^*^
Pre-test PSFS vs post-test PSFS	29	-17.17	5.78	-15.99	28	0.00^*^
Pre-test vs post-test muscle activation peak value	29	-80.05	100.65	-4.28	28	0.00^*^
Pre-test vs post-test muscle activation average value	29	-37.21	92.73	-2.16	28	0.03^*^

**Figure 1:**
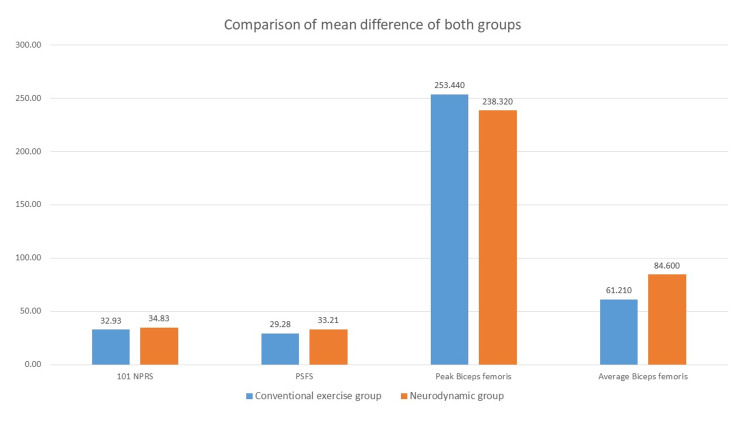
Comparison of mean difference in both groups NPRS, numeric pain rating scale; PSFS, patient-specific functional scale

The mean difference between the groups showed very little difference in improvements of the variables (Figure 1). Independent t-test of mean difference in between-group analysis showed insignificant (p>0.05) differences in 101 NPRS and EMG findings, This means there is no difference between both interventions regarding improvements in sciatic pain, and peak and average muscle activation of the biceps femoris muscle. Also, the result showed significant (p<0.05) differences in improvement in PSFS value (Table 4). This means that neurodynamic mobilization can improve the HRQL better.

**Table 4:** Comparison between the conventional and neurodynamic groups t, t-statistics; df, degree of freedom; f, f-statistics; p-value, probability value (95% confidence interval of the difference); NPRS, numeric pain rating scale; PSFS, patient-specific functional scale *p<0.05

Variables	N	Mean difference	t	df	f	P-value
101 NPRS	29	-1.90	-0.37	56	1.16	0.72
PSFS	29	-3.93	-2.53	56	0.43	0.01^*^
Muscle activation peak value	29	15.12	0.41	56	0.34	0.68
Muscle activation average value	29	-23.39	-1.15	56	4.36	0.25

The result suggests that conventional exercises can improve sciatica pain as well as peak muscle activation, which improves the HRQL in the patients. However, the average muscle activation does not improve significantly with conventional exercises, Whereas the neurodynamic mobilization of the sciatic nerve along with conventional exercises significantly improved the sciatica pain and both average and peak muscle activity; hence, the HRQL improved significantly in the patients. The comparative effect between the groups showed no significant difference between the improvement in sciatic pain and both peak and average muscle activation, but, still, the neurodynamic group has an upper hand in terms of HRQL over conventional exercises only due to the psychometric effect.

## Discussion

In this study, we found that conventional exercises had significantly reduced the pain and improved the HRQL of patients. The conventional exercises also improved the peak value of muscle activation of the biceps femoris, but the average value of muscle activation showed insignificant improvement. Studies showed that exercises targeted to improve the core muscles' performance help develop more stability in the lower back and hence cause influences that reduce radicular pain [13]. The authors also stated that the Golgi tendon organs present in the tendons fire when a muscle is contracted or stretched [14]. This leads to the spinal cord sending a signal to the agonistic muscles to relax, which may help the patient feel relaxed hence, feeling lesser pain, and having better HRQL. Simmonds also showed another angle of the placebo effect on reducing the pain and making the patient achieve better HRQL, which can vary from 7% to 72% on treatment [15]. Conventional exercises also increase blood circulation in the muscles around the spine and sciatic nerve, which enhances healing, and enhance activity, which increases muscle activation [5]. Exercise also helps in maintaining the normal length of surrounding muscles and soft tissues, for which we may have found the improvement in the peak muscle activation in EMG findings. Most studies that showed better muscle activation advocated long-term exercise plans. However, in this study, the effects of average muscle activation showed insignificant improvement, which is possibly due to the short-term exercise plan, which is not sufficient for improving the average effect.

On the other hand, the neurodynamic group in this study showed improvement in reducing pain, HRQL, and both peak and average muscle activation. Similar to this study, Geethika et al. found that neurodynamic mobilization along with conventional exercises can reduce LBP and lower extremity pain more than conventional exercises alone [16]. Cleland et al. explained that the compression of the sciatic nerve roots leads to the compromise of the microcirculation, and edema, and ultimately may cause demyelination of the sciatic nerve. Neurodynamic mobilization with oscillatory gliding movements can reduce this edema and reverse the hypoxia caused by compromised microcirculation. Hence, neurodynamic mobilization prevents the process of demyelination caused by sciatic nerve root compression [17]. The compression of the sciatic nerve root also causes adhesion of the nerve to its nerve sheath [18]. The gliding effect of neurodynamics causes the removal of adhesion and hence improves blood circulation and axonal transport, which is essential for maintaining the structural and functional integrity of the neuron [19]. Neurodynamic mobilization reduces the pressure causing intraneural and extra-neural fibrosis, thus increasing vascular and axoplasmic flow and restoring tissue mobility [20].

The comparison of both the groups, however, has not shown any significant difference except the improvement in HRQL. The possible reason for this may be the short duration of interventions. However, the improvements in both groups showed even short-duration effects of neurodynamic mobilization along with conventional exercises and conventional exercises alone. Sarkari and Multani also found similar findings in their study stating the combination of neural mobilization and exercise provided a better result in the range of SLR and LBP [21]; the finding of effects on HRQL, on the other hand, seems promising. The HRQL was measured using PSFS, which targets the patient's specificity of activities. It seems that due to the neurodynamic mobilization, the compressed sciatic nerve improves structurally and functionally. This helps better activation of the muscles. Thus, patients perform specific activities better than earlier. This makes the patient feel to have improved HRQL. Another possibility is that the psychological aspect of patients also improves when the fear of pain reduces and daily activities feel easy [3].

Limitations

This study was conducted for a short duration, that is, two weeks. No long-term follow-up was done. Further studies could be conducted by doing long-term follow-ups of the interventions. This may help us understand the long-duration effect of treatment on the patients as well as provide information on the recurrence of the symptoms.

## Conclusions

Radicular pain is reduced with both neurodynamics and conventional exercises. The immediate effects of the combination of neurodynamics along with conventional exercises showed better muscle activation than conventional exercises alone. As the symptoms improved, the HRQL of patients improved in both groups. Therefore, it can be concluded from this study that neurodynamics with conventional exercises can help in reducing pain, improving muscle activation of the biceps femoris, and elevating the HRQL of patients.
